# Targeting the regulation of aberrant protein production pathway in gastrointestinal cancer treatment

**DOI:** 10.3389/fonc.2022.1018333

**Published:** 2022-10-21

**Authors:** Hiromichi Sato, Kazuki Sasaki, Tomoaki Hara, Shogo Kobayashi, Yuichiro Doki, Hidetoshi Eguchi, Taroh Satoh, Hideshi Ishii

**Affiliations:** ^1^ Department of Gastrointestinal Surgery, Osaka University Graduate School of Medicine, Suita, Japan; ^2^ Department of Medical Data Science, Center of Medical Innovation and Translational Research, Osaka University Graduate School of Medicine, Suita, Japan

**Keywords:** SRP, RAPP, 7SL1, therapy, gastrointestinal cancer

## Introduction

Recent single-cell level sequence analysis in pancreatic cancer, which is a representative of intractable gastrointestinal cancers, revealed that the tumor tissue comprised of not only epithelial malignant cancer cells but also stromal activated fibroblasts and infiltrated immune cells. This indicates that various changes in gene expression occur in cells, including changes in cell-to-cell communication, to form a microenvironment that is characteristic of cancer (1–6). Epithelial malignant cancer cells harbor “big 4” driver mutations, that is, substitutions or alterations of nucleotides in KRAS proto-oncogene, GTPase (KRAS), tumor protein P53 (TP53), and cyclin-dependent kinase inhibitor 2A (CDKN2A), and mothers against decapentaplegic homolog 4 (SMAD4) commonly occurs in pancreatic ductal adenocarcinoma (PDAC) (https://portal.gdc.cancer.gov), which can be useful for predicting survival in patients with resected PDAC (7). Conversely, numerous gene expression alterations, due to cancer-specific transcription, RNA processing, and translations, are demonstrated by mesenchymal components, which include activated fibroblasts, vascular endothelial cells, and immune cells (8, 9). Importantly, this cancer-specific alterations induce the production of abnormal peptides and proteins with deleterious degenerations, which led to anti-apoptotic and pro-survival signals in cell-to-cell communication among tumor-component cells, contributing to biologically malignant phenotypes such as epithelial-to-mesenchymal transition phenotypes, invasion, and metastasis (8, 9). Thus, the aberrant protein production is important for the development of cancer-specific therapeutic approaches. Eventually, studies on mutation-prone tumors indicated that the mismatch-repair status predicted the clinical benefit of immune checkpoint blockade with anti-programmed cell death 1 reagents (10–12), suggesting that genetic mutations and resultant production of aberrant peptides or proteins may sensitize the response to cancer therapy. Recent studies have emerged that the importance of the regulation of aberrant protein production (RAPP) in many secretary and membrane proteins (13–16), playing a role in epithelial cancer cell and activated fibroblast or immune cell communication, the process associated with patient survival (6). In this article, we focused on the mechanism of RAPP in gastrointestinal cancer, especially pancreatic cancer, and explored the possibility of an innovative approach against intractable cancers.

## RAPP in cancer

The process of RAPP is mediated by a signal recognition particle (SRP), a ubiquitous initiator of protein translation (17), which is composed of six protein subunits arranged on a noncoding RNA, 7SL1 (16). The Alu domain is associated with SRP9 (9 kDa; encoded at the cytogenetic band 1q42.12) and SRP14 (14 kDa; encoded at the cytogenetic band 15q22) proteins, whereas the signal recognition domain is bounded by SRP19 (19 kDa; encoded at the cytogenetic band 5q22.2), SRP54 (54 kDa; encoded at the cytogenetic band 14q13.2), SRP68 (68 kDa; encoded at the cytogenetic band 17q25.1), and SRP72 (72 kDa; encoded at the cytogenetic band 4q12) proteins (16). Given that an SRP is a complex composed of multiple proteins encoded by multiple regions, it is suggested that it is responsible for a finely regulated mechanism (16, 17). This mechanism is also conserved across species, of which disruption can lead to various human diseases (16, 17). Here, we focused on the role of SRP in gastrointestinal cancers.

### SRP

Studies on the Alu-domain associated with SRP9 and SRP14 indicated that they are involved in several human cancers and can be used as diagnostic markers. The proteomic expression analysis of human colorectal cancer showed the upregulation of SRP9 with hypoxic adaptation of the tumor microenvironment of heterogeneous primary human tumor tissues (18), suggesting the role of SRP9 in RAPP in cancer. Interestingly, in adenosine-to-inosine RNA editing, 10 recurrent nonsynonymous RNA editing candidates were identified in nine genes, including the gene for SRP9 (19), indicating that the mechanism of those genes not directly encoded in the genomic DNA is implicated in colorectal cancer. The nucleotide sequence analysis of non-Hodgkin B-cell lymphoma, a hematopoietic malignancy, allowed the identification of an aberrant fusion gene of SRP9 conjoined with epoxide hydrolase 1 (EPHX1, encoded at the cytogenetic band 1q42.12) (20), suggesting that the mechanism that converts epoxides from the degradation of aromatic compounds to trans-dihydrodiols resulting in secretion from cells may be involved in this disease. The study of dysregulated RNA binding proteins identified 11 candidates of RNA binding proteins, including SRP14, which are involved in the hepatitis B virus (HBV)-related hepatocellular carcinoma prognosis (21), indicating that RAPP may be involved in the mechanism such as the HBV lifecycle and the progression of this disease. The study of pancreatic cancer PSN-1 cells indicated that the knockdown of SRP72 resulted in a significant increase in sensitization to radiation therapy as measured by colony formation assays, indicating that SRP72 is a marker of radiotherapy resistance against cancer (22).

### 7SL1

**Figure 1:**
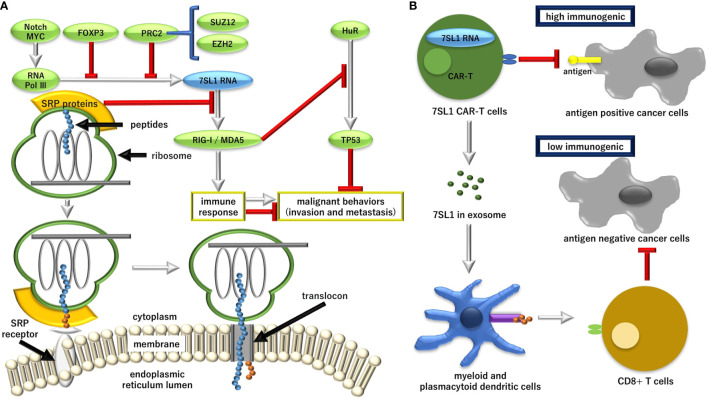
The role of endogenous immune stimulatory noncoding RNA 7SL1 in the pathway for the regulation of the aberrant protein production (RAPP) and its possible application in cancer therapy. **(A)** Transcriptional regulation of 7SL1 and the downstream mechanism for tumor suppressor tumor protein P53 (TP53). MYC, proto-oncogene, basic helix-loop-helix (BHLH) transcription factor (MYC); FOXP3, forkhead box P3; PRC2, polycomb repression complex 2; SUZ12, SUZ12 polycomb repressive complex 2 subunit; EZH2, enhancer of zeste 2 polycomb repressive complex 2 subunit; Pol II, RNA polymerase II; Pol III, RNA polymerase III; SRP, signal recognition particle; RIG-1, retinoic acid-inducible gene 1 protein; MDA5, melanoma differentiation-associated gene 5; HuR, embryonic lethal, abnormal vision, *Drosophila* (ELAV)-like 1, Hu antigen R. **(B)** Although the unshielded 7SL1-mediated stimulation of tumor tissues can result in disease progression, the exosomal delivery of 7SL1 or its combination with engineering chimeric antigen receptor (CAR)-T cells can stimulate an anti-tumor effect and lead to a favorable disease outcome. Ag, antigen.

Previous studies indicated that 7SL1, a noncoding RNA that binds to proteins to execute potent post-transcriptional regulation, is upregulated in cancer cells (23). It was demonstrated that 7SL1 binds to the 3’-untranslated region (UTR) of mRNA of tumor suppressor TP53 and that the interaction of 7SL1 with TP53 mRNA reduced the translation of p53 protein. On the other hand, the silencing of 7SL1 resulted in the increased binding of embryonic lethal, abnormal vision, *Drosophila* (ELAV)-like 1, Hu Antigen R (HuR) to TP53 mRNA, an interaction that led to the promotion of p53 translation, which reveals the competitive mechanism of 7SL1 and HuR for binding to TP53 3’-UTR (23). HuR selectively binds to adenylate-uridylate-(AU)-rich elements (AREs) found in the 3’-UTRs of various mRNAs. Given that AREs stimulate the degradation of mRNAs, HuR plays a role in stabilizing ARE-containing mRNAs by inhibiting degradation (24). The HuR is highly expressed in many cancers, including pancreatic cancer, functions as the post-transcriptional regulator of core metabolic enzymes, and is critical for survival under acute glucose deprivation in pancreatic cancer cells (25). In hepatocellular carcinoma, Wilms tumor 1-associating protein (WTAP) is significantly upregulated, and avian erythroblastosis virus E26 (V-Ets) oncogene homolog-1 (ETS1) was identified as the downstream effector of WTAP, suggesting that WTAP-guided m6A modification contributes to the progression of hepatocellular carcinoma *via* the HuR-ETS1-cyclin dependent kinase inhibitor 1A (p21)/cyclin dependent kinase inhibitor 1B (p27) axis (26). In pancreatic cancer, the role of mRNA modification at m6A in polo-like kinase 1 in cell cycle homeostasis was demonstrated (27), although the involvement of HuR and 7SL1 was elusive, suggesting that 7SL1 is involved, at least partially, in the process of antagonizing to p53 (**Figure 1**).

A study on forkhead box P3 (FOXP3), a transcription factor that is crucial for the development and inhibitory function of regulatory T-cells (Treg), indicated that overexpression of FOXP3 resulted in the repression of the transcription of 7SL1, whereas the knockdown of FOXP3 showed the upregulation of 7SL1 RNA transcription (28). The mechanism was confirmed by chromatin immuno-precipitation analysis and reporter assay (28). The study showed that FOXP3 promoted the expression of TP53 at the translational levels through repressing 7SL1 RNA (28). However, considering the recent study that indicated that Treg is chemoattracted to the tumor microenvironment by chemokine gradients such as C-C motif chemokine receptor 4 (CCR4)-C-C motif chemokine ligand 17 (CCL17)/CCL22, CCR8-CCL1, CCR10-CCL28, and CXCR3-CCL9/10/11, it is demonstrated that Treg cells are activated and inhibit antitumor immune responses, suggesting that strategies to deplete Treg cells and the control of Treg cell functions to increase antitumor immune responses are required in cancer immunotherapy (29), which demonstrates the multifaceted role of Treg in cancer. Given that FOXP3 unlikely represents the effect on 7SL1 exclusively in Treg cells, whether 7SL1 is an appropriate target to control Treg remains to be investigated.

Although a role of the polycomb repressive complex 2 (PRC2) component, working on the enhancer of zeste 2 polycomb repressive complex 2 subunit (EZH2)-mediated epigenetic control of RNA polymerase II (Pol II) transcribed coding gene transcription, has been well-established, a recent study on EZH2-mediated epigenetic regulation of RNA polymerase III (Pol III) transcription indicated that EZH2 is involved in the repression of Pol III transcription of tRNA(Tyr), 5S rRNA, and 7SL1 RNA genes *via* the interaction with transcriptional factor complex IIIC (TFIIIC) as well as SUZ12 polycomb repressive complex 2 subunit (SUZ12) (30), suggesting that basic mechanisms common to cells, which are specific mechanisms to cancer, are involved in 7SL1 regulation. Thus, it is necessary to study the upstream regulation of 7SL1 transcription in cells, which responds to various stimuli such as hypoxia and hyponutrition that are characterized by the extracellular tumor microenvironment.

## Damage-associated molecular patterns in cancer-associated fibroblasts-to-cancer cell interactions

In a single-cell sequence analysis (1–6), it was well-recognized that interactions between cancer cells and CAFs in stroma generate signals for cancer progression, inflammatory responses, and therapeutic outcomes (31–33). Previous reports have indicated that the arrangement of SRPs in 7SL1 is necessary to execute a physiological function of PARP in DAMPs (34). Reportedly, SRP dysfunction resulted in the induction of invasion and metastasis of cancer cells, which is associated with the evacuation from host’s immune response. A previous study elucidated that SRPs and 7SL1 are involved in the process of such signal, which revealed the importance of endogenous RNA response acting as damage-associated molecular patterns (35). In this study, it was demonstrated that triggering of notch receptor 1-MYC proto-oncogene and basic helix-loop-helix (BHLH) transcription factor (MYC) signaling in the stroma of breast cancer results in the activation of Pol III-driven increase in 7SL1, which is normally shielded by SRP9 and SRP 14 (35). The induced 7SL1 transcription resulted in an alteration of its stoichiometry with SRP9 and SRP 14, which led to the generation of unshielded 7SL1 in the stromal exosomes (35). Moreover, the unshielded 7SL1 can drive an inflammatory response. The unshielded 7SL1 activates the retinoic acid-inducible gene 1 protein (RIG-I), a pattern recognition receptor usually reserved for viral infections, to enhance tumor growth, metastasis, and therapy resistance, suggesting that the regulation of RNA unshielding can couple stromal activation with deployment of DAMPs of RNA, which is closely associated with the aggressive features of cancer (35). This also indicates the potential of 7SL1 as a target of cancer therapy, considering the immunogenic property of this endogenous non-coding RNA. The significant roles of exosome-mediated RNA transfer was demonstrated in a breast cancer study, indicating that CAFs orchestrate an intricate crosstalk with cancer cells by utilizing exosomes (36). The significance in gastrointestinal cancer warrants further investigation.

## Application to chimeric antigen receptor-T therapy

Recently, it was demonstrated that the immune stimulatory property can be utilized for the development of CAR-T cell therapy (37). The vector-mediated gene transfer of 7SL1 activated the RIG-I/melanoma differentiation-associated gene 5 (MDA5) signaling and promoted the expansion and effector-memory differentiation of CAR-T cells (37). MDA5 binds to RNAs with a modified DExD/H-box helicase core and a C-terminal domain, thus leading to a proinflammatory response that includes interferons (38, 39). RN7SL1 restricts myeloid-derived suppressor cell development, decreases transforming growth factor beta 1 (TGFB) in myeloid cells, and fosters dendritic cell subsets, which led to the endogenous expansion of effector-memory and tumor-specific T cells (37, 40). It is suggested that the unshielded form of 7SL1 can be used in peptide antigens to enhance anti-tumor efficacy (**Figure 1**).

## Discussion

Despite advances in surgery, standard radiation therapy, and chemotherapy, immunotherapy was added as a fourth treatment to cancer, as the discovery of immune modulation through immune check points and exploration of new combinations of cancer multimodality therapies led to overcoming long-term resistance and tumor recurrence (41). Cancer is a genetic disease involving numerous mutations (42–44), and the evidence that the mismatch-repair status predicted the clinical benefit of immune checkpoint blockade with pembrolizumab is important (10–12), as this suggests that the quantity or quality of gene mutations may influence susceptibility to immune checkpoint inhibitor therapy. Furthermore, the effect of immunotherapy can be maximized by regulating gene mutations and the consequent amino acid mutation neoantigen to eradicate cancer (45–47). If inducing a hot state in which cell-to-cell interactions are activated immunologically is possible, immune checkpoint inhibitors can not only be applied, but cancer-specific antigens can also be identified and used as vectors or employed as CAR-T therapy (48, 49). Nevertheless, methods to induce immunologically cold tumors into hot ones remain to be developed, and further investigations are necessary for application in a clinical setting.

The RAPP pathway study revealed the immune stimulatory property of endogenous non-coding RNA, 7SL1, which is a component of SRPs. A previous study suggests several important implications. First, given that the co-deploy peptide antigen with 7SL1 can exert an efficient anti-tumor effect (36), this approach may be useful to treat intractable solid tumors such as mutation-prone gastrointestinal cancer. Second, considering that unshielded 7SL1 in exosomes can be transferred in the tumor microenvironment (34), it is possible that the cell-to-cell communication is assessed by an RNA study in liquid biopsy as a companion diagnostic tool. Third, as radiotherapy can induce inflammatory microenvironment remodeling (50), the combination of RAPP pathway-mediated stimulatory cancer therapy with radiation may be useful to induce the immune response to convert an immune cold into a hot tumor, and multiple protocols have been developed (51). Fourth, nucleotide-mediated immune stimulation may be expanded as an anti-cancer therapy. Eventually, the intratumoral injection of the seasonal flu shot can generate systemic CD8+ T cell-mediated antitumor immunity and sensitizes resistant tumors to the checkpoint blockade, suggesting that it converts immunologically cold tumors to hot tumors and serves as an immunotherapy for cancer (52). To optimize the effect of treatment for intractable cancer, further studies to accumulate evidence are warranted.

## Author contributions

HS, KS, and HI conceptualized the study objectives and obtained the funding, wrote the manuscript. TH, SK, YD, HE, and TS outlined content of the manuscript. TS suggested which disease information should be addressed in the manuscript. All authors have read and approved the final manuscript version for publication.

## Funding

This work was supported in part by a Grant-in-Aid for Scientific Research from the Ministry of Education, Culture, Sports, Science and Technology (19K22658; 20H00541; 22H03146; 22K19559), and Japan Agency for Medical Research and Development (AMED) (17cm0106414h0002; JP19lm0203007; JP20lm0203007; JP21lm0203007). Partial support was received from Princess Takamatsu Cancer Research Fund and Mitsubishi Foundation to HI.

## Acknowledgments

Authors are thankful to every lab member.

## Conflict of interest

Partial institutional endowments were received from Taiho Pharmaceutical Co., Ltd. (Tokyo, Japan), Hirotsu Bio Science Inc. (Tokyo, Japan); Kinshu-kai Medical Corporation (Osaka, Japan); Kyowa-kai Medical Corporation (Osaka, Japan); IDEA Consultants Inc. (Tokyo, Japan); Unitech Co. Ltd. (Chiba, Japan).

The remaining authors declare that the research was conducted in the absence of any commercial or financial relationships that could be construed as a potential conflict of interest.

## Publisher’s note

All claims expressed in this article are solely those of the authors and do not necessarily represent those of their affiliated organizations, or those of the publisher, the editors and the reviewers. Any product that may be evaluated in this article, or claim that may be made by its manufacturer, is not guaranteed or endorsed by the publisher.
